# Surveillance of Tick-Borne Pathogens (TBPs) from Wildlife in the Valle d’Aosta Region, Northwestern Italy

**DOI:** 10.3390/pathogens15090999

**Published:** 2026-09-21

**Authors:** Chiara Nogarol, Serena Robetto, Nicoletta Vitale, Raffaella Spedicato, Santina Di Bella, Cristina Panteghini, Beatrice Vuillermoz, Nadège Haudemand, Riccardo Orusa, Maria Lucia Mandola

**Affiliations:** 1 Istituto Zooprofilattico Sperimentale del Piemonte, Liguria e Valle d’Aosta, 10154 Torino, Italy; serena.robetto@izsplv.it (S.R.); nicoletta.vitale@izsplv.it (N.V.); raffaella.spedicato@izsplv.it (R.S.); beatrice.vuillermoz@izsplv.it (B.V.); nadege.haudemand@izsplv.it (N.H.); marialucia.mandola@izsplv.it (M.L.M.); 2 National Reference Centre for Anaplasma, Babesia, Rickettsia and Theileria (C.R.A.Ba.R.T.), 90129 Palermo, Italy; santina.dibella@izssicilia.it (S.D.B.); cristina.panteghini@izssicilia.it (C.P.); 3 National Reference Centre for Wild Animal Diseases (CeRMAS), 11020 Aosta, Italy

**Keywords:** ticks, TBPs, wildlife, zoonosis, Alps

## Abstract

Tick-borne diseases are emerging worldwide due to the expanding distribution of tick vectors and represent an increasing public health concern. This study investigated the occurrence of major tick-borne pathogens in ticks collected from wild animals in Valle d’Aosta Region (Northwestern Italy) between 2017 and 2022. Tick pools were screened for *Anaplasma* spp., *Borrelia burgdorferi* s.l., *Coxiella burnetii*, *Rickettsia* spp., and Tick-Borne Encephalitis virus (TBEV). Most collected ticks were morphologically identified as *Ixodes ricinus*. Overall, 15.2% of the 158 analyzed pools tested positive for at least one pathogen. *Rickettsia* spp. was the most frequently detected pathogen (10.7%), with multiple species identified, including *R. helvetica*, *R. monacensis*, *R. slovaca*, and *R. raoultii*. *Anaplasma phagocytophilum* was detected in ticks collected from wild ungulates, whereas *Borrelia burgdorferi* s.l. was only occasionally identified. No samples tested positive for TBEV or *C. burnetii*. *Rickettsia* spp. positivity was higher in ticks collected from ungulates and in *I. ricinus*, although these differences were not statistically significant. These findings provide new insights into tick ecology in Alpine ecosystems, highlighting the circulation of potentially zoonotic pathogens and the altitudinal presence of ticks in the Alps up to 1800–2300 m a.s.l. The study contributes valuable epidemiological data and supports the need for continued surveillance within a One Health framework.

## 1. Introduction

Ticks are obligate hematophagous ectoparasites of animals and humans and their life cycle and related distribution are dependent on several factors like climate, host availability and anthropogenic factors [1]. Warmer temperatures have been shown to have positive effects on the phenology of exophilic tick vectors, such as *Ixodes ricinus* (*I. ricinus*), especially on the limits of their latitudinal and altitudinal range [2], promoting tick dispersion and establishment in new geographic areas. The rise of vector populations and the growing evidence of pathogens they may carry is a deeply studied topic even if many aspects require a more thorough investigation [3].

Wild animals are recognized as reservoirs of several zoonotic pathogens: they could serve as amplifying hosts for both pathogens and vectors and/or may act as mechanical transport of ticks even over long distances [4]. In Europe, the most important and widespread three active life stages (larva, nymph, adult) tick vector is *I. ricinus* [5,6] which is characterized by relative lack of host specificity, with more than 300 vertebrate species documented as hosts of them [7] especially at the larval and nymph stage. Small- to medium-sized mammals, reptiles and birds [8] are recognized as the most frequent host and adult female of *I. ricinus* feed only on large and medium-sized mammals with deer representing the most important animals for the maintenance of the local tick population [9]. It should be noted that several other ungulate species are feeding and propagation hosts for the tick *I. ricinus* as well as hosts to a wide range of zoonotic pathogens and are often considered as sentinels of flaviviruses and tick-borne zoonotic pathogen circulation [3,10]. The relative contribution to the life cycle of *I. ricinus* and the transmission cycles of tick-borne pathogens differ among the ungulate species [11].

The most prevalent zoonotic agents transmitted by this species of tick belong to the *Borrelia burgdorferi* sensu lato complex [12]. *I. ricinus* may also be considered reservoir hosts of *Anaplasma phagocytophilum* (*A. phagocytophilum*) [13] and may act as both a vector and reservoir for *Rickettsia* spp. [11]. *Coxiella burnetii* (*C. burnetii*) has been identified in *I. ricinus*, although its vector capacity is still debated for this pathogen [14,15]. The detection of TBE virus in ticks is challenging and time-consuming since the prevalence is restricted to small foci of viral circulation [16,17] also including new neurotropic tick-borne flavivirus subtype [18].

Considering the climatic conditions, *I. ricinus* is susceptible to high temperatures and very dry environments because they are not conducive to its survival [5]; conversely, countries with shorter summers and longer winters are well suited to the survival of this species [19]. Ixodid ticks are ectotherms, and therefore their life cycle is influenced by weather conditions, particularly temperature and relative humidity. In general, a daily maximum temperature of 10 °C can be considered the minimum threshold for Ixodid ticks’ activity, while 35 °C represents the critical threshold. Rising temperatures could increase the likelihood of host-seeking activity but also expose ticks to a higher risk of mortality. At the same time, relative humidity influences tick water balance, thereby affecting host-seeking behavior and survival [20]. Climatic conditions above a certain altitude also limit the spread of *I. ricinus*, although recent observations in Europe and Italy have shown that its presence is increasingly detected at higher altitudes, at an up to 1650 m above sea level [21].

Well-organized surveillance system on the prevalence of tick-borne diseases is lacking, even if the incidence is increasing in several European regions due to better climate suitability, as well as in the Northern part of Italy [22,23]. In particular, the incidence of human tick-borne diseases (TBDs) in non-endemic Italian areas is certainly underestimated, due to limited surveillance (both at human and veterinary levels), asymptomatic cases, false negative results in serological tests and an inadequate number of follow up studies [24].

A previous study conducted by part of the same research team, published by Guardone et al. [6], applied a similar methodological approach to investigate the presence of ticks and tick-borne pathogens (TBPs) in the Liguria Region. However, the objectives of the present study are not merely confirmatory. Liguria and Valle d’Aosta differ substantially in their geographical, climatic, ecological, and wildlife characteristics. Valle d’Aosta is a predominantly Alpine region, whereas Liguria extends from the Apennines to the Mediterranean coast. These environmental differences may affect both tick species composition and TBPs circulation patterns. Consequently, findings obtained in Liguria cannot be directly extrapolated to Valle d’Aosta. The present study was therefore designed to generate region-specific data on tick occurrence and TBPs in wildlife in Valle d’Aosta (a mountainous autonomous region in northwestern Italy, located between the major Alpine massifs and bordering France to the west and Switzerland to the north), where information remains scarce. One of the main objectives of this study is therefore to contribute to filling the existing knowledge gap by providing a comprehensive overview of tick occurrence and TBPs in wildlife across the region. This information is particularly relevant given the widespread distribution of wildlife and the frequent occurrence of both occupational and recreational activities in natural and rural environments, which may increase opportunities for human exposure to tick vectors.

## 2. Materials and Methods

### 2.1. Study Area

The Valle d’Aosta region, located in Northwestern Italy, is the country’s smallest region and has an approximately rectangular shape, extending about 80 km in length and 40 km in width. It borders the Piedmont Region to the east and south, Switzerland to the north, and France to the west. The territory is predominantly mountainous, covering a surface area of 3260.8 km^2^, with a mean elevation of 2106 m above sea level. Altitude ranges from approximately 310 m in the southeastern part of the region to 4810 m at Mont Blanc, with more than 60% of the territory lying above 2000 m. Nestled among the major Alpine massifs, the region is crossed only in its southeastern sector by the Dora Baltea River, which flows toward the Canavese plain. This distinctive orographic configuration has preserved much of the territory in a natural state: approximately 40% consists of rocky or glacial surfaces, 51% is covered by forests and pastures, and only 9%—mainly located along the central valley floor and side valleys—is suitable for human settlements and agricultural activities, hosting a population of around 123,000 inhabitants [25].

The Valle d’Aosta region is characterized by a semi-continental alpine climate, with cold, snowy winters and mild, relatively dry summers in the lower valleys, gradually transitioning to arctic conditions at higher elevations. Vegetation changes markedly with altitude, ranging from vineyards and chestnut woodlands below 800 m, to red spruce and larch forests on intermediate slopes, and alpine grasslands with dwarf shrubs above 2000 m. The Alps form a barrier that makes inland areas drier, resulting in wetter, colder conditions at higher elevations and valleys with lower precipitation extending down to the lower Dora Baltea area, which is characterized by a typical Mediterranean climate.

### 2.2. Tick Collection and Identification

Ticks were collected from wild animals between 2017 and 2022 as part of the regional wildlife monitoring and passive surveillance activity implemented by the Valle d’Aosta Region. It should be noted that the hunting season, as defined by regional regulations, differs among species following the Regional Wildlife and Hunting Plan (https://www.regione.vda.it/risorsenaturali/Fauna_selvatica/attivita_venatoria_i.aspx, accessed on 1 September 2026), which is the main legal framework governing wildlife management, conservation, and hunting of Valle d’Aosta region. It also manages selective culling and conservation measures for local alpine wildlife, including chamois, deer, and wild boar.

Moreover, the Regional Wildlife Health Monitoring Plan represents a structured wildlife health surveillance program covering the main wildlife species present in the regional territory. Species-specific sampling targets are defined according to the abundance/density of the different wildlife populations in the region. The Regional Wildlife Health Monitoring Plan is not specifically designed for entomological surveillance. Therefore, although wildlife sampling covers the entire regional territory according to the targets established by the Plan, tick collection reflects the animals examined within this surveillance framework and is influenced by host species, hunting seasons, carcass availability, and tick infestation. Accordingly, the sampling design was intended to provide information on the occurrence and distribution of ticks associated with monitored wildlife and on the occurrence of tick-borne pathogens in the collected ticks, rather than to estimate tick abundance or pathogen prevalence in the environmental tick population.

Ticks were removed by Ce.R.M.A.S. veterinary staff from hunted wild game, including wild boar (*Sus scrofa*), red deer (*Cervus elaphus*), roe deer (*Capreolus capreolus*), chamois (*Rupicapra rupicapra*) or from wild animals found dead and delivered by foresters to the IZSPLV for necropsy such as Alpine ibex (*Capra ibex*), red fox (*Vulpes vulpes*), European badger (*Meles meles*), wolf (*Canis lupus*), beech marten (*Martes foina*), hedgehog (*Erinaceus europaeus*), and squirrel (*Sciurus vulgaris*) [26]. Animals were systematically inspected for the presence of ticks: all carcasses were inspected with protective gloves and ticks were removed from the feeding sites using fine pointed tweezers. Ticks were collected up to 3–6 h (depending on the host species) after host killing and quickly were put in labeled tubes with 70% ethanol. Ticks were subsequently morphologically identified under a stereomicroscope to genus/species level, life stage, and sex according to the identification keys described by Estrada-Peña et al. [27]. All ticks collected from the same animal and belonging to the same species and life stage were pooled (mean number of ticks per pool = 3; range: 1–5) and preserved in sample vials with 70% ethanol until molecular analysis (Section 2.3). In this study, with the exception for two pools of different species removed from the same roe deer, one pool corresponds to one animal.

### 2.3. Molecular Analysis for Pathogen Detection

Tick homogenates were prepared from either (i) engorged female ticks (one tick was longitudinally cut in two equal parts using sterile forceps and surgical blades and one half was used for nucleic acid extraction), (ii) non-engorged adults or (iii) pools of up to 4 nymphs and/or larvae (grouped according to species, development stage, gender and host). The mechanical disruption was carried out with Tissue Lyser (TissueLyser II, QIAgen, Germany) and ceramic beads, followed by the extraction of nucleic acid with Maxwell^®^ RSC viral TNA Kit procedure (Promega Corporation, Madison, WI, USA). Details of target genes and primers sequences used for pathogen screening are listed in Table 1. All the positive controls used in the PCRs for molecular screening of TBPs were reference materials; in particular, the positive control for *C. burnetii* is an inactivated culture from ANSES, Maisons-Alfort, and the TBE-positive control comes from an inactivated cell culture from Istituto Zooprofilattico Sperimentale delle Venezie. All the positive inactivated cultures were extracted with the same procedure applied to the samples. The PCR methods were previously described and used also in Guardone et al. [6].

### 2.4. Molecular Confirmation and Sequencing of Anaplasma spp. and Rickettsia spp.

Nucleic acids extracted from samples testing positive during the initial tick-borne pathogen (TBP) screening (Section 2.3) were submitted to the National Reference Centre for Anaplasma, Babesia, Rickettsia and Theileria (C.R.A.Ba.R.T.), for confirmatory molecular analyses and species identification. To confirm *Rickettsia* spp. DNA, conventional PCR assays were conducted targeting the *ompA*, *ompB*, and *gltA* genes, with species-level identification carried out using Sanger sequencing of the *ompB* amplicon. For *Anaplasma* spp., confirmatory PCR and species identification were performed by amplification and sequencing of the 16S rRNA gene. PCR reactions were carried out in a final volume of 50 μL containing 5 μL of template DNA and GoTaq^®^ G2 DNA Polymerase (Promega Italia s.r.l., Milan, Italy). DNA from Rickettsia conorii (Amplirun, Vircell, Granada, Spain) and Anaplasma phagocytophilum extracted from IFA slides (Fuller Laboratories, Fullerton, CA, USA) were included as positive controls, whereas nuclease-free water served as the negative control. PCR products were visualized on 2% agarose gels. Amplicons showing the expected size and sufficient amplification intensity were purified and subjected to Sanger sequencing (Macrogen Europe, Amsterdam, The Netherlands). Samples showing insufficient amplification intensity were not subjected to sequencing. Sequence chromatograms were inspected and edited using BioEdit v7.7 (Tom Hall, Ibis Biosciences, Carlsbad, CA, USA), and consensus sequences were compared with reference sequences available in the NCBI GenBank database using the Basic Local Alignment Search Tool (on-line BLAST version). Species identification was based on the closest matching reference sequences obtained by BLAST. The BLAST identification percentage and the country of origin of the reference sequence used for species assignment are reported in Supplementary Table S1.

### 2.5. Data Analysis

A comprehensive dataset including sampling date, host species, municipality of origin, altitude, tick species, and pathogen detection results was filled out and subjected to statistical analysis. The number of tick pools collected from each host category and the proportion of pathogen-positive pools were calculated. To account for ecological differences among wildlife hosts, host species were grouped into ungulates (wild boar, roe deer, red deer, chamois, and Alpine ibex) and non-ungulates wild species. Tick species were grouped into *Ixodes ricinus* group and other tick species. Seasons were classified as spring–summer and autumn–winter according to the expected seasonal activity of ticks in alpine environments, while altitude was categorized into three ecological classes (<500 m, 500–1000 m, and >1000 m above sea level).

Given the limited number of pathogen-positive pools and the sparse distribution of positive observations across several categories of the investigated factors, statistical analyses were primarily descriptive and exploratory. Pathogens with few positive detections were described by reporting positivity proportions and corresponding exact 95% binomial confidence intervals. Inferential analyses were restricted to *Rickettsia* spp., as it was the only pathogen with a sufficient number of positive pools to allow exploratory comparisons across the investigated factors. Associations between *Rickettsia* spp. positivity and host group, tick group, season, and altitude class were assessed using Fisher’s exact test, as appropriate for small and sparse contingency tables. Multivariable logistic regression was also explored, but the available data did not support reliable estimation of independent effects; therefore, univariate results were retained and interpreted as exploratory associations. Pathogen-specific positivity was calculated as the proportion of positive pools among those examined in each category. In the case of co-detection of multiple pathogens, the same pool contributed to the positivity estimate of each pathogen detected. A *p*-value < 0.05 was considered statistically significant. All statistical analyses were performed using RStudio (R software environment, version 2024.04.2+764), while QGIS was used for spatial visualization and mapping of tick species and pathogen distribution.

## 3. Results

Table 2 describes the number of collected pools of ticks. The pools were grouped for tick species, host and percentage of each tick species over the total number of pools collected for that host. Table S2 of Supplementary Materials describes for each host–tick combination, the number of ticks examined (N), and the number (n) and percentage (%) of ticks positive for each pathogen with the corresponding 95% confidence interval (95% CI). All the positive pools were sent to the National Reference Centre for *Anaplasma*, *Babesia*, *Rickettsia*, e *Theileria* (C.R.A.Ba.R.T.) for confirmatory PCR analyses and sequencing. The number of examined hosts and association between host and tick was assessed by Fisher’s exact test. Statistically significant associations are shown by *p*-value calculated using Fisher’s exact test within the subset of ungulates/non-ungulates wild species.

Out of the 158 tick pools collected, 127 were obtained from wild ungulates. Specifically, 76 pools were collected from roe deer, 25 from chamois, 19 from red deer, 6 from wild boar, and 1 from Alpine ibex. The most represented tick genus/species was *Ixodes* spp./*I. ricinus* (*n* = 120; 94.5%), whereas only 7 pools (5.5%) belonged to *Dermacentor* spp./*D. marginatus.* A total of 23 out of 127 tick pools (18%) tested positive for at least one target pathogen at the S.S. Virologia Specialistica (IZSPLV Torino). The identified pathogens, together with the associated tick and host species, are reported in Table 3. The most frequently detected pathogen genus was *Rickettsia* spp., identified in 16 tick pools. All *Rickettsia* spp.-positive pools were subjected to confirmatory PCR assays targeting the *ompA*, *ompB*, and *gltA* genes. Among the 17 *Rickettsia* spp.-positive pools, 15 yielded *ompB* amplicons with sufficient amplification intensity for Sanger sequencing and subsequent species-level identification. Sequence analysis identified four *Rickettsia* species, namely *R. helvetica*, *R. monacensis*, *R. slovaca*, and *R. raoultii*. The remaining two positive pools were reported as *Rickettsia* spp. Concerning the association between *Rickettsia* species, tick species, and vertebrate hosts, most positive ticks belonged to *I. ricinus* collected from roe deer, red deer, and chamois. The most frequently detected species was *R. helvetica*, identified in 9 out of 16 positive tick pools collected from roe deer and red deer, followed by *R. monacensis*, detected in 4 out of 16 positive pools sampled from roe deer, red deer, and chamois. *R. raoultii* was detected in only one tick pool collected from a red deer, while only one *D. marginatus* pool, collected from a roe deer, tested positive for *R. slovaca*. *Anaplasma phagocytophilum* was detected in 5 out of 16 positive tick pools, specifically in pools collected from roe deer, red deer, and chamois. Finally, only two tick pools tested positive for *Borrelia burgdorferi* s.l., both collected from roe deer; interestingly, these two pools were co-infected with *R. helvetica*.

Out of the 158 tick pools collected, 31 were obtained from non-ungulates wild species. Tick pools were collected from 13 red foxes, 9 European badgers, 4 wolves, 2 beech martens, 2 hedgehogs, and 1 squirrel. All the pools were morphologically identified as *Ixodes* spp./ *I. ricinus*, whereas only 1 tick was identified as *Rhipicephalus sanguineus*. Only one pool, consisting of *I. ricinus* collected from a European badger, tested positive for *Rickettsia* spp. (Table 3). No tick pools tested positive for Tick-Borne Encephalitis (TBE) virus and *C. burnetii*.

**Figure 1:**
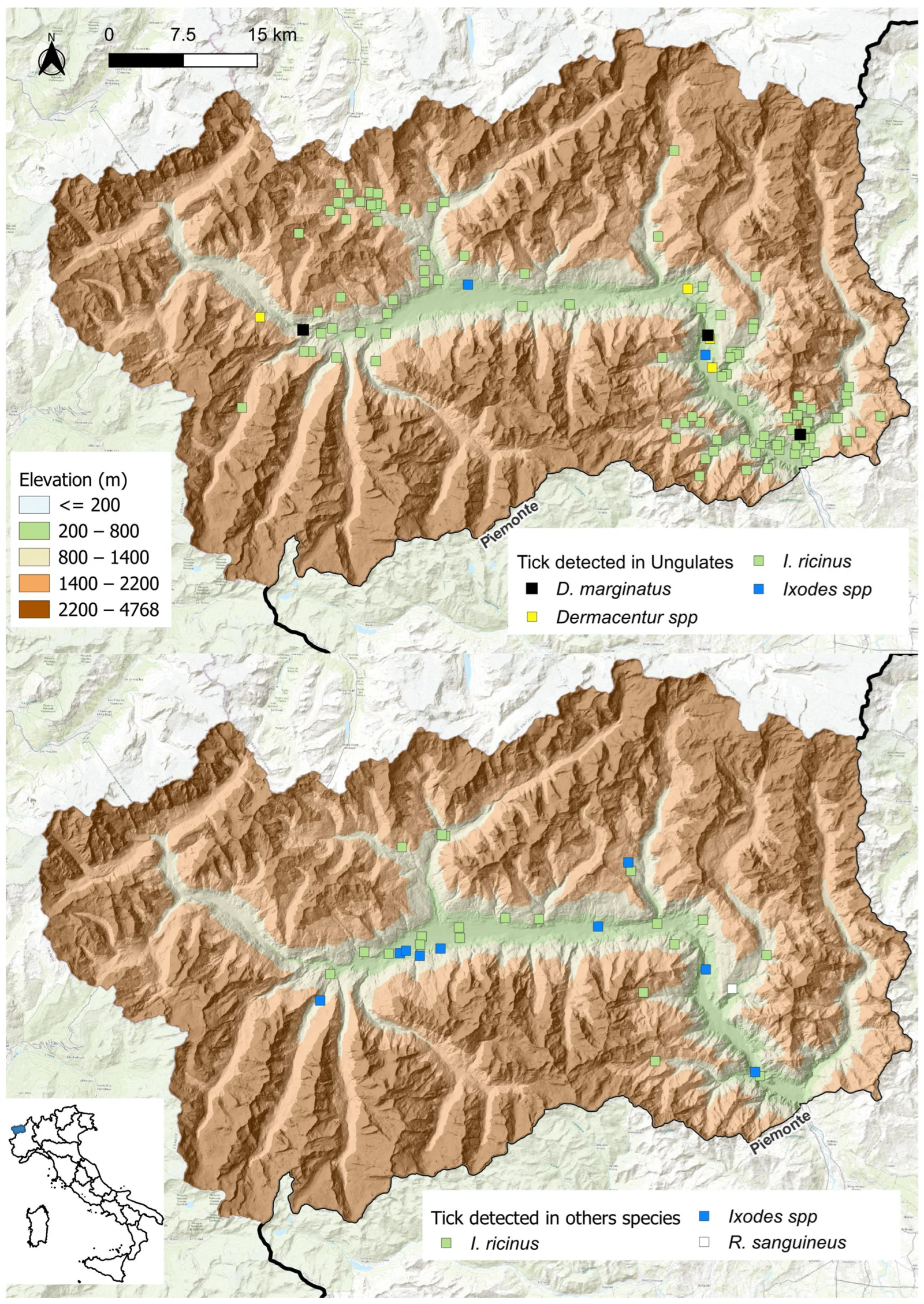
Spatial distribution of tick species detected in wildlife. The upper Valle d’Aosta map shows altimetric levels in morphological format according to the number of tick pools species in ungulates. The lower Valle d’Aosta map shows altimetric levels in morphological format according to the number of tick pools species in non-ungulates species. The inset map indicates the location of the study area within Italy.

Figure 1 describes the distribution of tick species correlated to the pathogen positivity and the distribution along the sampled territory. Ticks’ sampling was mainly concentrated in municipalities located along the central valley corridor, where most pathogen-positive pools were also detected. *Ixodes ricinus* represented the dominant tick species across the study area, while *Dermacentor* spp. and *Rhipicephalus sanguineus* were detected only occasionally. Positive pools for *Rickettsia* spp. were widely distributed, whereas *Anaplasma* spp. detections were less frequent and spatially more localized.

**Figure 2:**
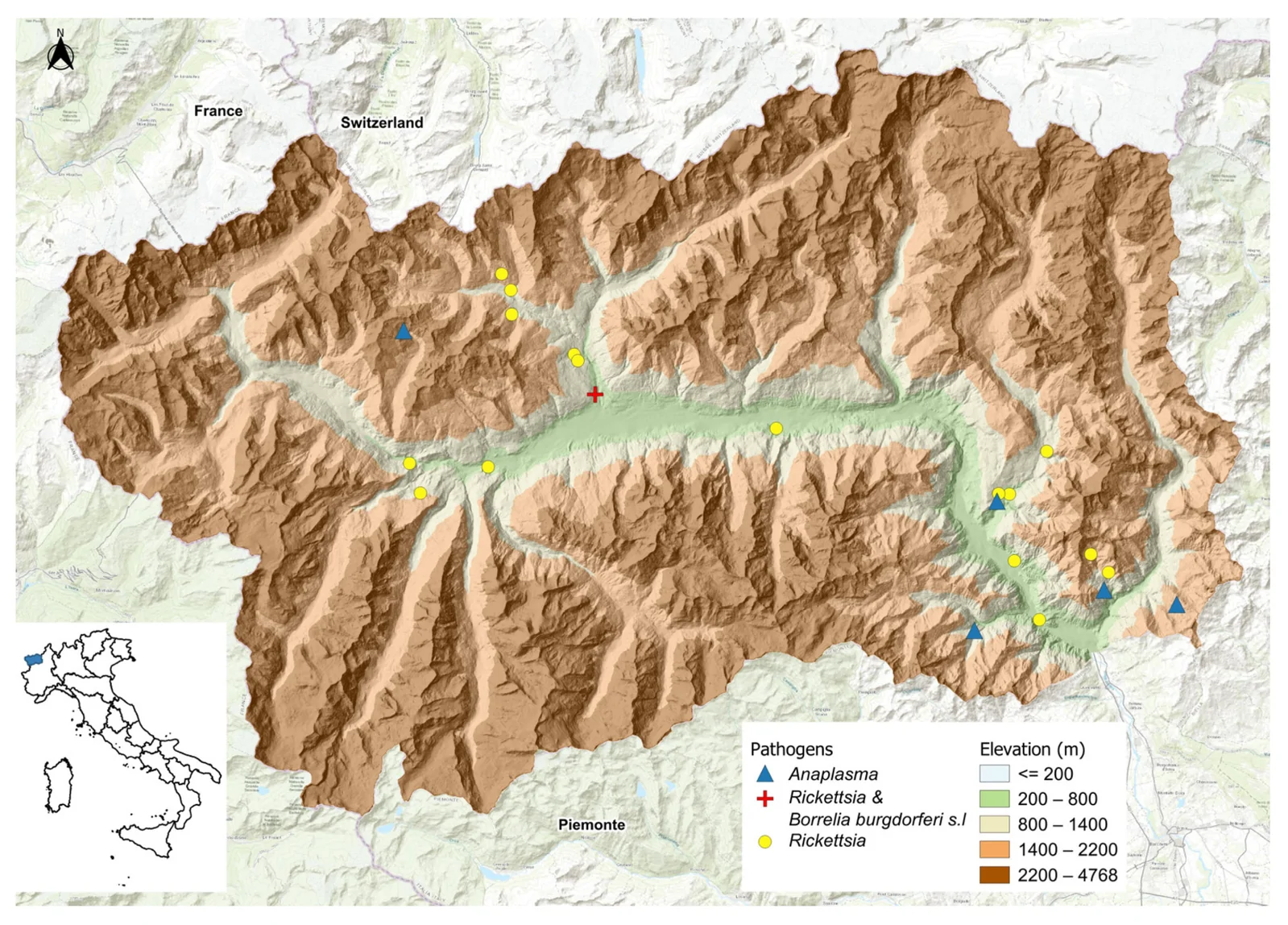
Spatial distribution of tick-borne pathogens detected in wildlife ticks. Sampling locations positive for *Rickettsia* spp. are shown in yellow, locations positive for *Anaplasma* spp. in blue, and sites with co-detection of *Borrelia burgdorferi* s.l. and *Rickettsia* spp. in red. The inset map indicates the location of the study area within Italy.

The spatial distribution of tick-borne pathogens detected in wildlife ticks collected in the Valle d’Aosta region is detailed in Figure 2. Most positive samples for *Rickettsia* spp. were detected in low and medium altitude areas distributed along the main valley axis, whereas *Anaplasma* spp.-positive samples were less frequent and more spatially scattered. Table 3 reports data about each pathogen detection in association to tick and host species, including the elevation at which host animals were sampled.

The prevalence of *Rickettsia* spp.-positive pools (Table 4) was higher in ticks collected from ungulates (13.4%) compared with other wild species (3.2%), although the association was not statistically significant (Fisher’s exact test, *p* = 0.2). Similarly, *Ixodes ricinus* showed a higher prevalence of *Rickettsia* spp. positivity (12.2%) compared with other tick species (5.3%), but no significant association was observed (*p* = 0.7). A significant seasonal difference was detected, with a higher prevalence of *Rickettsia* spp.-positive pools during the spring–summer period (22.0%) compared with autumn–winter (7.0%) (Fisher’s exact test, *p* = 0.006). No significant association was observed between altitude class and *Rickettsia* spp. positivity (*p* = 0.57), although the highest prevalence was observed at altitudes below 500 m (20.0%).

## 4. Discussion

It is well-established that wildlife plays a critical role in tick epidemiology, serving both as a primary host and as a reservoir for tick-borne pathogens. Wildlife directly provides blood meals necessary for tick growth and reproduction and contributes to the dispersal of ticks and pathogens through both natural movements (e.g., avian migration) and human-facilitated movements (e.g., wildlife trade) [28]. The present study provides new insights into the presence of tick-borne pathogens in the Valle d’Aosta region (Northwestern Italy), an area for which detailed information on the occurrence of ticks carrying zoonotic agents, their association with wildlife species, and their distribution across different altitudes and habitats has not been updated to date. We observed that the genus/species *Ixodes* spp./*I. ricinus* accounted for 94.5% of all ticks collected from wild ungulates (Table 2), consistent with findings from other studies conducted in both Europe and Italy, where this genus/species is particularly abundant in pre-alpine and hilly areas of northern regions [5,6,29,30,31,32]. Recent studies have also highlighted increasing population densities and demographic expansion of some ungulate species, particularly roe deer and red deer [33,34,35], as well as Alpine ibex [32]. This may create favorable conditions for the spread of *I. ricinus* populations towards higher elevations, increasing contact with host populations that were previously marginally exposed to these ticks. Regarding wild boar, the present study revealed a comparable occurrence of *Ixodes* and *Dermacentor* tick genera (Table 2). A strong host–parasite association between wild boar and adult *D. marginatus* has already been documented in Mediterranean regions [6,26,36,37,38,39]. The distribution of this species is primarily driven by its preference for warm and dry (xerophilic) environments, particularly shrublands, pastures, and the edges of deciduous forests [40]. Consequently, habitat overlaps between wild boar and *Ixodes* spp./*Dermacentor* spp. may enhance the epidemiological role of wild boar in the maintenance and transmission of tick-borne epizootic and zoonotic pathogens [40,41]. Similarly to wild ungulates, *Ixodes* spp. was the predominant tick genus found on wild carnivores and mesocarnivores, accounting for 97% of all collected ticks (Table 2). *Ixodidae* was the only tick family detected on both red foxes and wolves, in agreement with findings reported from other northwestern Italian regions [26]. The study also showed that European badgers, hedgehogs, and squirrels were parasitized exclusively by *Ixodidae* ticks. These species represent important hosts in the life cycle of hard ticks, particularly in peri-urban environments, where they may act as bridging hosts facilitating the circulation of tick-borne pathogens at the wildlife–urban interface [42]. No other tick species were detected, except for a single *Rhipicephalus sanguineus* collected from a beech marten. This finding represents an uncommon infestation, as this tick species exhibits a strong host preference for dogs, which serve as the primary host, only occasionally parasitizing wild carnivores and humans. A few cases of human parasitism by *R. sanguineus* ticks have been described in the literature; however, its ecological adaptability suggests that the risk of human parasitism may increase in areas experiencing warmer temperatures and/or prolonged summer seasons [43].

In Italy, data on tick abundance have traditionally been reported at elevations up to approximately 1300 m above sea level [37]. This altitudinal threshold is generally associated with lower temperatures and more open habitats, characterized by less favorable microclimatic conditions and reduced host availability for tick survival and development [37,44]. However, in the western Italian Alps, the occurrence of both *I. ricinus* and *D. marginatus* at elevations above 1000 m has been increasingly documented [32,45,46].

The present study provides evidence of presence of ticks in Alpine environments up to 1800–2300 m a.s.l. (Table 3). This finding is particularly relevant, as it documents the occurrence of *I. ricinus* at high elevations and is consistent with previous studies suggesting that its distribution may be influenced by multiple interacting ecological and environmental factors, including availability of mammalian hosts. Moreover, over the last ten years, average temperatures in the Valle d’Aosta region have been rising at all elevations. The zero-degree isotherm is rising significantly; consequently, the altitude of the freezing level during summer peaks has at times exceeded 4500 m. Furthermore, although total annual precipitation has seen a slight decrease in some areas, rainfall intensity has increased, leading to a high risk of flash floods and sudden intense downpours. These changes in regional climate and environmental conditions may be considered important if we consider that we found infested hosts at 1800–2300 m a.s.l. All these factors may contribute to an increased risk of tick exposure for both humans and wildlife at higher elevations, as stated in Garcia-Vozmediano et al., 2020 [46]: the presence of *I. ricinus* in the Alpine arch, also in the Western Italian side, represent evidence on the shift towards a new suitable habitat for *Ixodes* ticks.

Regarding pathogens, 15.2% of the tested pools were positive for at least one pathogen, confirming findings from studies conducted in northeastern Italy [47] and Sicily [48], which reported similar positivity rates in ticks collected from tick-bitten humans (15.4%), environmental samples, and wild boars (14%). Conversely, the positivity rates observed in our study were lower than those reported in studies conducted on ticks collected from wild ungulates in northwestern and northeastern Italy [6,49]. A higher proportion of *Rickettsia* spp.-positive pools was observed during spring–summer (22.0%) than during autumn–winter (7.0%). Although this pattern is consistent with the greater biological activity of ticks under favorable climatic conditions [50], it should be interpreted cautiously, as the exploratory univariate analysis does not allow an independent seasonal effect to be established. The overall prevalence of *Rickettsiae* detected in our study (10.7%, 17/158) was comparable to that reported by Scarpulla et al. [51] (12.4%) in 113 ticks collected from hosts and the environment in the Latium and Tuscany regions, but lower than the prevalences reported by Ebani et al. [52] (20.78%) and Pascucci et al. [53] (52.25%) in 178 pools of *Ixodes* ticks collected from wild species in the Abruzzo region. Data on Valle d’Aosta region are reported in Millet et al. [54], where *Rickettsia* spp. prevalence was assessed at 13.3% and *B. burgdorferi* s.l. prevalence was 40.0%, even if these data are collected from dragging strategy and not from wildlife. Comparisons among studies should be interpreted with caution, as differences among tick sampling and pooling strategies may affect the comparability of the results. Several limitations should also be considered, including the passive surveillance approach adopted which was restricted to wildlife hosts and therefore influenced by seasonal patterns and hunting activities, as well as the relatively small number of samples analyzed (158 pools). These limitations should be considered when interpreting prevalence estimates. Nevertheless, the long duration of the survey and the broad geographical coverage of the sampling area represent important strengths of the study. The primary objective of this study was to provide updated information on the presence of tick’s species and the occurrence of TBPs in a region for which published data are scarce and have not been updated in recent years, especially from animals. Although the limited sample size may have affected the estimation of a pathogen prevalence, the data generated provides a valuable baseline for future studies investigating tick distribution and its potential association with climatic and environmental variables. Finally, certain methodological and molecular limitations of our screening approach should be acknowledged. While standard molecular assays combined with Sanger sequencing successfully achieved robust species-level identification (as detailed in Supplementary Table S1), the targeted diagnostic markers utilized exhibit a high degree of sequence conservation. Consequently, these markers are primarily validated for reliable species confirmation rather than high-resolution intra-species discrimination. Furthermore, the primary objective of this surveillance study was to establish an epidemiological baseline of pathogen occurrence and geographic distribution across wildlife interfaces in the Alpine region rather than an exhaustive evolutionary characterization of individual strains. Future investigations incorporating multi-locus markers are warranted to assess whether the detected pathogens represent single clonal strains or multiple genetically distinct lineages co-circulating in this ecosystem.

Sequence analysis of *Rickettsia* spp.-positive pools identified four different species, with *R. helvetica* being the most prevalent, followed by *R. monacensis*, *R. slovaca*, and *R. raoultii*. The diversity of *Rickettsia* spp. detected in our study is consistent with findings reported across Italy in recent decades, largely owing to advances in molecular diagnostic techniques [48,55,56]. Our results showed a strong association between *R. helvetica* and roe deer, supporting the possible role of wild ruminants, including roe deer, red deer, and chamois, as feeding hosts for *Ixodidae* ticks as well as contributors to the maintenance and circulation of bacterial pathogens in wildlife ecosystems [6,10]. *R. helvetica* is an emerging human pathogen belonging to the spotted fever group (SFG) *Rickettsiae*: human infections have been reported in France, Switzerland, and Italy and are generally characterized by mild, self-limiting illness associated with fever, headache, and myalgia, usually in the absence of cutaneous rash [57,58]. Human infections caused by *R. monacensis* have been reported in both Italy [59] and elsewhere in Europe, although the number of documented cases remains low, possibly because this species is considered only mildly pathogenic and often causes self-limiting infections [58,60]. In our study, *R. monacensis* was detected in *I. ricinus* in two pools collected from roe deer and one pool collected from red deer; *R. slovaca* and *R. raoultii* were each detected in a single pool, respectively, from a *D. marginatus* from roe deer and from an *I. ricinus* from red deer. These findings further support the well-established association between *R. slovaca* and *D. marginatus*, although the prevalence observed in our study was lower than that reported elsewhere [56,61]. Both bacterial species are recognized etiological agents of scalp eschar and neck lymphadenopathy after tick bite (SENLAT). Nevertheless, diagnosis of SENLAT is still frequently based on clinical and epidemiological findings alone, without serological or molecular confirmation [62].

Five *I. ricinus* pools tested positive for *Anaplasma phagocytophilum*, representing 20.8% of all TBP-positive pools. Positive samples originated from two roe deer, one red deer, and two chamois. Notably, the positive pool collected from red deer originated from an animal sampled at 2300 m a.s.l., documenting the occurrence of *I. ricinus* carrying *A. phagocytophilum* at high altitude. Although *A. phagocytophilum* is frequently detected in animals and ticks, human cases are comparatively rare in Europe. This may be explained by the existence of distinct genetic variants, only some of which are associated with human disease. Lesiczka et al. [63] identified a zoonotic subcluster of *A. phagocytophilum* associated with dogs, horses, cats, carnivores, wild boar, and hedgehogs.

*B. burgdorferi* s.l. was detected in two *I. ricinus* pools collected from roe deer. In both cases, the pools were also positive for *R. helvetica*, representing the only co-detections identified in this study. Coinfections involving multiple TBPs within the same tick have been widely documented worldwide, both in ticks collected from wildlife and in questing or engorged ticks, sometimes reaching different prevalence values [49,64,65]. In particular, the presence of one or more coinfecting pathogens during Lyme disease may exacerbate clinical manifestations [66] and represent an important concern in endemic areas, especially among occupationally exposed individuals [67]. Data on Lyme disease in humans were reported by Trevisan et al. [68], who documented only three confirmed cases in the Valle d’Aosta region during the observation period from 2010 to 2022. Regarding human rickettsioses, data covering the period from 1996 to 2010 were published by the Istituto Superiore di Sanità [69], reporting three confirmed cases in the Valle d’Aosta region. To the best of our knowledge, no other recent reports or scientific publications providing updated data on the diagnosis of tick-borne pathogens in humans are currently available for this region. However, comparisons between human and tick surveillance data should be interpreted with caution, as the diagnosis of TBDs in humans relies primarily on serological testing, which may differ substantially from the molecular methods used for pathogen detection in ticks. Finally, neither *C. burnetii* nor TBEv were detected in the pools analyzed in this study. In particular, the absence of TBEv is consistent with the current epidemiological situation in Italy, where the virus is mainly reported in the northeastern regions of the country [17], with only sporadic cases documented in northwestern areas [18].

## 5. Conclusions

Risk characterization in northwestern Italian territories is of particular importance considering the underreporting of human cases in this part of the Alpine arch in contrast to the strategic geographical position of this area, located at the crossroads between the Mediterranean Sea, Western Europe, and northeastern Italy. Further investigations in the Valle d’Aosta region are therefore warranted, preferably integrating machine learning approaches to identify and predict areas at increased risk of tick-borne diseases. Such tools could support the development of more targeted and effective prevention and control strategies, optimizing the allocation of public health resources. Targeted awareness campaigns directed at local communities, together with enhanced preparedness measures encompassing both prevention and response, through an approach that also incorporates “citizen science” could contribute substantially to reducing the public health risks associated with exposure to tick-borne pathogens [70].

In this context, a One Health strategy integrating human, animal, and environmental data would represent a valuable approach for the early detection and mitigation of emerging tick-borne disease threats in Alpine ecosystems.

## Figures and Tables

**Table 1:** Primers and target gene for the PCR protocols used for tick-borne pathogens (TBPs) screening.

Pathogen	Primers	Target Gene
*Anaplasma* spp.	16SANA-F 5′-CAGAGTTTGATCCTGGCTCAGAACG-3′ 16SANA-R 5′-GAGTTTGCCGGGACTTCTTCT GTA-3′	*16S rRNA*
*Borrelia burgdorferi* s.l.	FLA1 5′-AGAGCAACTTACAGACGAAATTAAT-3′ FLA2 5′-CAAGTCTATTTTGGAAAGCACCTAA-3′	*FLA*
*Coxiella burnetii*	Trans1 5′-TATGTATCCACCGTAGCCAG C-3′ Trans2 5′-CCCAACAACACCTCCTTATTC-3′	*IS1111*
*Rickettsia* spp.	RpCS.877p 5′-GGGGGCCTGCTCACGGCGG-3′ RpCS.1258n 5′-ATTGCAAAAAGTACAGTGAACA-3′	*Citrate synthase*
Tick-Borne Encephalitis (TBE) virus	F-TBE 5′-GGGCGGTTCTTGTTCTCC-3′ R-TBE 5′-ACACATCACCTCCTTGTCAGACT-3′ TBE-Probe-WTTGAGCCACCATCACCCAGACACA	*3′ non-coding region*

**Table 2:** Number of the tick pools collected for each host species and percentage of each tick species over the total number of ticks collected for that host species; n = number of examined hosts and association between host and tick assessed by Fisher’s exact test. Statistically significant associations are shown by * *p*-value calculated using Fisher’s exact test within the subset of ungulates/non-ungulates wild species. *ns p* > 0.05, * *p* ≤ 0.05, ** *p* < 0.001.

	Ungulates Species						Non-Ungulates Wild Species						
Tick Species	Roe Deer (*n* = 75)	Chamois (*n* = 25)	Red Deer (*n* = 19)	Wild Boar (*n* = 6)	Alpine Ibex (*n* = 1)	Overall (*n* = 126)	Red Fox (*n* = 13)	European Badger (*n* = 9)	Wolf (*n* = 4)	Beech Marten (*n* = 2)	Hedgehog (*n* = 2)	Squirrel (*n* = 1)	Overall (*n* = 31)
***Ixodes* spp. **	2 (2.6%)	0	0	0	0	2 (1.6%)	5 (38.5%)	3 (33.3%)	0	0	0	1(100.0%)	9 (29%)
***I. ricinus***	71 (93.4%)	25 (100.0%)	19 (100.0%)	2 (33.3%) **	1 (100.0%)	118 (92.9%)	8 (61.5%)	6 (66.7%)	4 (100.0%)	1 (50.0%)	2 (100.0%)	0	21 (67.7%)
***Dermacentor* spp. **	2 (2.6%)	0	0	2 (33.3%) *	0	4 (3.1%)							
***D. marginatus***	1 (1.3%)	0	0	2 (33.3%) *	0	3 (2.4%)							
***R. sanguineus***							0	0	0	1 (50.0%) *	0	0	1 (3.0%)
**Overall number of collected tick pools**	76	25	19	6	1	127	13	9	4	2	2	1	31

**Table 3:** Details of the identified pathogens in relation to tick species and host (the elevation at which host animals were sampled is also reported) and number of pools confirmed positive.

Identified Pathogen	Host Species	Tick Species	Number of Confirmed Positive Pools (PCR and Sequencing)	Altitudes of Host Species (m a.s.l)
*Anaplasma phagocytophilum*	Roe deer	*I. ricinus*	2	800 m, 1700 m
	Red deer	*I. ricinus*	1	2300 m
	Chamois	*I. ricinus*	2	1200 m
*Borrelia burgdorferi* s.l. *	Roe deer	*I. ricinus*	2*	700 m
*Rickettsia* spp.	Roe deer	*I. ricinus*	1	750 m
	European badger	*I. ricinus*	1	1000 m
*R. raoultii*	Red deer	*I. ricinus*	1	1760 m
*R. slovaca*	Roe deer	*D. marginatus*	1	800 m
*R. monacensis*	Roe deer	*I. ricinus*	2	300 m, 1300 m
	Red deer	*I. ricinus*	1	1320 m
	Chamois	*I. ricinus*	1	450 m
*R. helvetica*	Roe deer	*I. ricinus*	8	480 m, 580 m 700 m 800 m (2 hosts) 1000 m, 1300 m 1800 m
	Red deer	*I. ricinus*	1	1800 m

* both co-infected with *R. helvetica*.

**Table 4:** Univariate analysis of factors associated with *Rickettsia* spp. positivity in tick pools collected from wildlife hosts in the Aosta Valley region (Northwestern Italy). The table reports the number of tested pools, prevalence of *Rickettsia* spp. positive pools by host group, tick species, season, and altitude class, together with Fisher’s exact test results and corresponding *p*-values.

Factors	Category	n° Pool	*Rickettsia* spp. Prevalence	Test	*p* Value
Host	Ungulates	127	13.40%	Fisher	0.2
	Non-ungulates	31	3.20%		
Tick species	*Ixodex ricinus*	139	12.20%	Fisher	0.7
	other ticks species	19	5.30%		
Season	Spring–Summer	50	22.00%	Fisher	0.006
	Autumn–Winter	108	7.00%		
Altitude	<500 m	15	20.00%	Fisher	0.57
	500–1000 m	64	10.90%		
	>1000 m	79	10.10%		

## Data Availability

The original contributions presented in this study are included in the article; further inquiries can be directed at the corresponding authors.

## Supplementary Materials

The following supporting information can be downloaded at https://www.mdpi.com/article/10.3390/pathogens15090999/s1, Table S1. Molecular identification of *Rickettsia* spp. and *Anaplasma* spp. based on Sanger sequencing and BLAST analysis; Table S2. Detection of tick-borne pathogens according to tick and host species. For each host–tick combination, the number of ticks examined (N), and the number (n) and percentage (%) of ticks positive for each pathogen with the corresponding 95% confidence interval (95% CI) are reported. Percentages were calculated using the number of ticks examined within each host–tick combination as denominator. In case of co-infections, the same tick is included in each relevant pathogen-specific column; therefore, pathogen-specific positive counts are not mutually exclusive and should not be summed.

## References

[1] Stroffolini G., Segala F.V., Lupia T., Faraoni S., Rossi L., Tomassone L., Zanet S., De Rosa F.G., Di Perri G., Calcagno A. (2021). Serology for *Borrelia* spp. in Northwest Italy: A Climate-Matched 10-Year Trend. Life.

[2] Jore S., Viljugrein H., Hofshagen M., Brun-Hansen H., Kristoffersen A.B., Nygård K., Brun E., Ottesen P., Sævik B.K., Ytrehus B. (2011). Multi-source analysis reveals latitudinal and altitudinal shifts in range of *Ixodes ricinus* at its northern distribution limit. Parasites Vectors.

[3] Grassi L., Drigo M., Zelená H., Pasotto D., Cassini R., Mondin A., Franzo G., Tucciarone C.M., Ossola M., Vidorin E. (2023). Wild ungulates as sentinels of flaviviruses and tick-borne zoonotic pathogen circulation: An Italian perspective. BMC Vet. Res..

[4] Tomassone L., Berriatua E., De Sousa R., Duscher G.G., Mihalca A.D., Silaghi C., Sprong H., Zintl A. (2018). Neglected vector-borne zoonoses in Europe: Into the wild. Vet. Parasitol..

[5] Gray J., Kahl O., Zintl A. (2024). Pathogens transmitted by Ixodes ricinus. Ticks Tick Borne Dis..

[6] Guardone L., Nogarol C., Accorsi A., Vitale N., Listorti V., Scala S., Brusadore S., Miceli I.N., Wolfsgruber L., Guercio A. (2024). Ticks and Tick-Borne Pathogens: Occurrence and Host Associations over Four Years of Wildlife Surveillance in the Liguria Region (Northwest Italy). Animals.

[7] Gray J., Kahl O., Zintl A. (2021). What do we still need to know about *Ixodes ricinus*?. Ticks Tick Borne Dis..

[8] Boulanger N., Aran D., Maul A., Camara B.I., Barthel C., Zaffino M., Lett M.C., Schnitzler A., Bauda P. (2024). Multiple factors affecting *Ixodes ricinus* ticks and associated pathogens in European temperate ecosystems (northeastern France). Sci. Rep..

[9] Kahl O., Gray J.S. (2023). The biology of *Ixodes ricinus* with emphasis on its ecology. Ticks Tick Borne Dis..

[10] Kazimírová M., Hamšíková Z., Špitalská E., Minichová L., Mahríková L., Caban R., Sprong H., Fonville M., Schnittger L., Kocianová E. (2018). Diverse tick-borne microorganisms identified in free-living ungulates in Slovakia. Parasites Vectors.

[11] Fabri N.D., Sprong H., Hofmeester T.R., Heesterbeek H., Donnars B.F., Widemo F., Ecke F., Cromsigt J.P.G.M. (2021). Wild ungulate species differ in their contribution to the transmission of Ixodes ricinus-borne pathogens. Parasites Vectors.

[12] Wolcott K.A., Margos G., Fingerle V., Becker N.S. (2021). Host association of *Borrelia burgdorferi* sensu lato: A review. Ticks Tick. Borne Dis..

[13] Grassi L., Franzo G., Martini M., Mondin A., Cassini R., Drigo M., Pasotto D., Vidorin E., Menandro M.L. (2021). Ecotyping of *Anaplasma phagocytophilum* from wild ungulates and ticks shows circulation of zoonotic strains in northeastern Italy. Animals.

[14] Špitalská E., Sparagano O., Stanko M., Schwarzová K., Zdenko Å., Škultéty Ľ., Havlíková S.F. (2018). Diversity of *Coxiella*-like and *Francisella*-like endosymbionts, and *Rickettsia* spp., *Coxiella burnetii* as pathogens in the tick populations of Slovakia, Central Europe. Ticks Tick Borne Dis..

[15] Knap N., Žele D., Glinšek Biškup U., Avšič-Županc T., Vengušt G. (2019). The prevalence of *Coxiella burnetii* in ticks and animals in Slovenia. BMC Vet. Res..

[16] Gaffuri A., Sassera D., Calzolari M., Gibelli L., Lelli D., Tebaldi A., Vicari N., Bianchi A., Pigoli C., Cerioli M. (2024). Tick-Borne Encephalitis, Lombardy, Italy. Emerg. Infect. Dis..

[17] Da Rold G., Obber F., Monne I., Milani A., Ravagnan S., Toniolo F., Sgubin S., Zamperin G., Foiani G., Vascellari M. (2022). Clinical Tick-Borne Encephalitis in a Roe Deer (*Capreolus capreolus* L.). Viruses.

[18] Nowotny N., Mandola M.L., Monne I., Bagó Z., Nogarol C., Fusaro A., Dimmel K., Moroni B., Guardone L., Kolodziejek J. (2025). Neurotropic Tick-Borne Flavivirus in Alpine Chamois (*Rupicapra rupicapra rupicapra*), Austria, 2017, Italy, 2023. Viruses.

[19] Gray J.S., Kahl O., Robertson J.N., Daniel M., Estrada-Peña A., Gettinby G., Jaenson T.G., Jensen P., Jongejan F., Korenberg E. (1998). Lyme borreliosis habitat assessment. Zentralblatt Bakteriol..

[20] Ogden N.H., Lindsay L.R. (2016). Effects of Climate and Climate Change on Vectors and Vector-Borne Diseases: Ticks Are Different. Trends Parasitol..

[21] Martello E., Mannelli A., Ragagli C., Ambrogi C., Selmi M., Ceballos L.A., Tomassone L. (2014). Range expansion of *Ixodes ricinus* to higher altitude, and co-infestation of small rodents with *Dermacentor marginatus* in the Northern Apennines, Italy. Ticks Tick Borne Dis..

[22] Alfano N., Tagliapietra V., Rosso F., Ziegler U., Arnoldi D., Rizzoli A. (2020). Tick-borne encephalitis foci in northeast Italy revealed by combined virus detection in ticks, serosurvey on goats and human cases. Emerg. Microbes Infect..

[23] Beltrame A., Rodari P., Mauroner L., Zanella F., Moro L., Bertoli G., Da Re F., Russo F., Napoletano G., Silva R. (2021). Emergence of Lyme borreliosis in the province of Verona, Northern Italy: Five-years of sentinel surveillance. Ticks Tick Borne Dis..

[24] Otranto D., Dantas-Torres F., Giannelli A., Latrofa M.S., Cascio A., Cazzin S., Ravagnan S., Montarsi F., Zanzani S.A., Manfredi M.T. (2014). Ticks infesting humans in Italy and associated pathogens. Parasites Vectors.

[25] ISTAT. http://dati.istat.it/.

[26] Accorsi A., Schiavetti I., Listorti V., Dellepiane M., Masotti C., Ercolini C., Guardone L., Razzuoli E. (2022). Hard Ticks (*Ixodidae*) from Wildlife in Liguria, Northwest Italy: Tick Species Diversity and Tick-Host Associations. Insects.

[27] Estrada-Peña A., Bouattour A., Camicas J.L., Walker A.R. (2004). Ticks of Domestic Animals in the Mediterranean Region: A Guide to Identification of Species.

[28] Tsao J.I., Hamer S.A., Han S., Sidge J.L., Hickling G.J. (2021). The Contribution of Wildlife Hosts to the Rise of Ticks and Tick-Borne Diseases in North America. J. Med. Entomol..

[29] Sevestre J., Diarra A.Z., Oumarou H.A., Durant J., Delaunay P., Parola P. (2021). Detection of emerging tick-borne disease agents in the Alpes-Maritimes region, southeastern France. Ticks Tick Borne Dis..

[30] Perez G., Bournez L., Boulanger N., Fite J., Livoreil B., McCoy K.D., Quillery E., René-Martellet M., Bonnet S.I. (2023). The distribution, phenology, host range and pathogen prevalence of *Ixodes ricinus* in France: A systematic map and narrative review. Peer Community J..

[31] Bertola M., Montarsi F., Obber F., Da Rold G., Carlin S., Toniolo F., Porcellato E., Falcaro C., Mondardini V., Ormelli S. (2021). Occurrence and Identification of *Ixodes ricinus* Borne Pathogens in Northeastern Italy. Pathogens.

[32] Menzano A., Tizzani P., Farber M.D., Garcia-Vozmediano A., Martinelli L., Rossi L., Tomassone L. (2024). Zoonotic Tick-Borne Pathogens in Ticks from Vegetation and Alpine Ibex (*Capra ibex*) in the Maritime Alps, Italy. Animals.

[33] De Pasquale D., Dondina O., Scancarello E., Meriggi A. (2019). Long-term viability of a reintroduced population of roe deer *Capreolus capreolus,* in a lowland area of northern Italy. Folia Zool..

[34] Kiffner C., Lödige C., Alings M., Vor T., Rühe F. (2010). Abundance estimation of *Ixodes* ticks (Acari: *Ixodidae*) on roe deer (*Capreolus capreolus*). Exp. Appl. Acarol..

[35] Tagliapietra V., Rosà R., Arnoldi D., Cagnacci F., Capelli G., Montarsi F., Hauffe H.C., Rizzoli A. (2011). Saturation deficit and deer density affect questing activity and local abundance of *Ixodes ricinus* (Acari, *Ixodidae*) in Italy. Vet. Parasitol..

[36] Sgroi G., Iatta R., Lia R.P., D’Alessio N., Manoj R.R.S., Veneziano V., Otranto D. (2021). Spotted fever group rickettsiae in *Dermacentor marginatus* from wild boars in Italy. Transbound. Emerg. Dis..

[37] Medlock J.M., Hansford K.M., Bormane A., Derdakova M., Estrada-Peña A., George J.C., Golovljova I., Jaenson T.G., Jensen J.K., Jensen P.M. (2013). Driving forces for changes in geographical distribution of *Ixodes ricinus* ticks in Europe. Parasites Vectors.

[38] Satta G., Chisu V., Cabras P., Fois F., Masala G. (2011). Pathogens and symbionts in ticks: A survey on tick species distribution and presence of tick-transmitted micro-organisms in Sardinia, Italy. J. Med. Microbiol..

[39] Grech-Angelini S., Stachurski F., Vayssier-Taussat M., Devillers E., Casabianca F., Lancelot R., Uilenberg G., Moutailler S. (2020). Tick-borne pathogens in ticks (Acari: *Ixodidae*) collected from various domestic and wild hosts in Corsica (France), a Mediterranean island environment. Transbound. Emerg. Dis..

[40] Selmi M., Tomassone L., Ceballos L.A., Crisci A., Ragagli C., Pintore M.D., Mignone W., Pautasso A., Ballardini M., Casalone C. (2018). Analysis of the environmental and host-related factors affecting the distribution of the tick *Dermacentor marginatus*. Exp. Appl. Acarol..

[41] Hrazdilová K., Lesiczka P.M., Bardoň J., Vyroubalová Š., Šimek B., Zurek L., Modrý D. (2021). Wild boar as a potential reservoir of zoonotic tick-borne pathogens. Ticks Tick Borne Dis..

[42] Prandi I., Serrano E., Maas M., Fonville M., Wattimena A., Quaranta G., Capucchio M.T., Sprong H., Tomassone L. (2026). Single and Co-Infections by Tick-Borne Pathogens in Synanthropic European Hedgehogs (*Erinaceus europaeus*) in Northwestern Italy. Vet. Sci..

[43] Parola P., Socolovschi C., Jeanjean L., Bitam I., Fournier P.E., Sotto A., Labauge P., Raoult D. (2008). Warmer Weather Linked to Tick Attack and Emergence of Severe Rickettsioses. PLoS Negl. Trop. Dis..

[44] Estrada-Peña A. (2001). Distribution, abundance, and habitat preferences of *Ixodes ricinus* (Acari: *Ixodidae*) in northern Spain. J. Med. Entomol..

[45] Pintore M.D., Ceballos L., Iulini B., Tomassone L., Pautasso A., Corbellini D., Rizzo F., Mandola M.L., Bardelli M., Peletto S. (2015). Detection of Invasive *Borrelia burgdorferi* Strains in North-Eastern Piedmont, Italy. Zoonoses Public Health.

[46] Garcia-Vozmediano A., Krawczyk A.I., Sprong H., Rossi L., Ramassa E., Tomassone L. (2020). Ticks climb the mountains: Ixodid tick infestation and infection by tick-borne pathogens in the Western Alps. Ticks Tick Borne Dis..

[47] Moro L., Da Rold G., Beltrame A., Formenti F., Mazzi C., Ragusa A., Scarso S., Drigo I., Degani M., Piubelli C. (2025). Surveillance of Tick-Borne Pathogens in Ticks from Humans in the Province of Verona, Italy (2018–2022): A Prospective Study. Microorganisms.

[48] Di Bella S., Blanda V., Scibetta S., Giacchino I., Gentile A., Chiarenza G., Cannella V., Provinzano G., Grippi F., Guercio A. (2024). Molecular Detection of *Rickettsia* spp. and Other Tick-Borne Pathogens in Ticks from a Nature Reserve: Implications for Zoonotic Transmission. Animals.

[49] Rosso F., Ferrari G., Weil T., Tagliapietra V., Marini G., Dagostin F., Arnoldi D., Girardi M., Rizzoli A. (2024). Temporal Changes in Tick-Borne Pathogen Prevalence in Questing *Ixodes ricinus* Across Different Habitats in the North-Eastern Italian Alps. Microbiologyopen.

[50] ECDC. https://www.ecdc.europa.eu/en/news-events/reducing-risk-tick-borne-encephalitis-summer.

[51] Scarpulla M., Barlozzari G., Marcario A., Salvato L., Blanda V., De Liberato C., D’Agostini C., Torina A., Macrì G. (2016). Molecular detection and characterization of spotted fever group rickettsiae in ticks from Central Italy. Ticks Tick Borne Dis..

[52] Ebani V.V., Bertelloni F., Turchi B., Filogari D., Cerri D. (2015). Molecular survey of tick-borne pathogens in Ixodid ticks collected from hunted wild animals in Tuscany, Italy. Asian Pac. J. Trop. Med..

[53] Pascucci I., Cammà C. (2010). Lyme disease and the detection of *Borrelia burgdorferi* genospecies in *Ixodes ricinus* ticks from central Italy. Vet. Ital..

[54] Millet I., Ragionieri M., Tomassone L., Trentin C., Mannelli A. (2019). Assessment of the Exposure of People to Questing Ticks Carrying Agents of Zoonoses in Aosta Valley, Italy. Vet. Sci..

[55] Guccione C., Colomba C., Iaria C., Cascio A. (2023). Rickettsiales in the WHO European Region: An update from a One Health perspective. Parasites Vectors.

[56] Pascucci I., Antognini E., Canonico C., Montalbano M.G., Necci A., di Donato A., Moriconi M., Morandi B., Morganti G., Crotti S. (2021). One Health Approach to Rickettsiosis: A Five-Year Study on Spotted Fever Group *Rickettsiae* in Ticks Collected from Humans, Animals and Environment. Microorganisms.

[57] Fournier P.E., Allombert C., Supputamongkol Y., Caruso G., Brouqui P., Raoult D. (2004). Aneruptive fever associated with antibodies to *Rickettsia helvetica* in Europe and Thailand. J. Clin. Microbiol..

[58] Parola P., Paddock C.D., Socolovschi C., Labruna M.B., Mediannikov O., Kernif T., Abdad M.Y., Stenos J., Bitam I., Fournier P.E. (2014). Update on tick-borne rickettsioses around the world: A geographic approach. Clin. Microbiol. Rev..

[59] Madeddu G., Fiore V., Mancini F., Caddeo A., Ciervo A., Babudieri S., Masala G., Bagella P., Nunnari G., Rezza G. (2016). Mediterranean spotted fever-like illness in Sardinia, Italy: A clinical and microbiological study. Infection.

[60] Jado I., Oteo J.A., Aldámiz M., Gil H., Escudero R., Ibarra V., Portu J., Portillo A., Lezaun M.J., García-Amil C. (2007). *Rickettsia monacensis* and human disease, Spain. Emerg. Infect. Dis..

[61] Maioli G., Pistone D., Bonilauri P., Pajoro M., Barbieri I., Mulatto P., Vicari N., Dottori M. (2012). Ethiological [corrected] agents of rickettsiosis and anaplasmosis in ticks collected in Emilia-Romagna region (Italy) during 2008 and 2009. Exp. Appl. Acarol..

[62] Barlozzari G., Romiti F., Zini M., Magliano A., De Liberato C., Corrias F., Capponi G., Galli L., Scarpulla M., Montagnani C. (2021). Scalp eschar and neck lymphadenopathy by *Rickettsia slovaca* after *Dermacentor marginatus* tick bite case report: Multidisciplinary approach to a tick-borne disease. BMC Infect. Dis..

[63] Lesiczka P.M., von Loewenich F.D., Kohl R., Krawczyk A.I., Dirks R.P., Boyer P.H., Jaulhac B., Moniuszko-Malinowska A., Uršič T., Strle F. (2025). European human granulocytic anaplasmosis is caused by a subcluster of *Anaplasma phagocytophilum* Ecotype I. Curr. Res. Parasitol. Vector Borne Dis..

[64] Borşan S.D., Ionică A.M., Galon C., Toma-Naic A., Peştean C., Sándor A.D., Moutailler S., Mihalca A.D. (2021). High Diversity, Prevalence, and Co-infection Rates of Tick-Borne Pathogens in Ticks and Wildlife Hosts in an Urban Area in Romania. Front. Microbiol..

[65] Raileanu C., Moutailler S., Pavel I., Porea D., Mihalca A.D., Savuta G., Vayssier-Taussat M. (2017). *Borrelia* Diversity and Co-infection with Other Tick-Borne Pathogens in Ticks. Front. Cell. Infect. Microbiol..

[66] Popov G., Bashchobanov D., Andonova R. (2026). Tick-Borne Co-Infection in Lyme Disease: Clinical Impact, Diagnostic Challenges, and Therapeutic Perspectives. Microorganisms.

[67] Stinco G., Bergamo S. (2015). Impact of Co-Infections in Lyme Disease. Open Dermatol. J..

[68] Trevisan G., Ruscio M., Cinco M., Nan K., Forgione P., Di Meo N., Tranchini P., Nacca M., Trincone S., Rimoldi S.G. (2023). The history of Lyme disease in Italy and its spread in the Italian territory. Front. Pharmacol..

[69] https://www.iss.it/documents/20126/45616/onlinegenn_2015.pdf/cba73700-e3c5-c18e-c976-433ae510e2bf?t=1581097449388.

[70] Sgroi G., Iatta R., Lia R.P., Napoli E., Buono F., Bezerra-Santos M.A., Veneziano V., Otranto D. (2022). Tick exposure and risk of tick-borne pathogens infection in hunters and hunting dogs: A citizen science approach. Transbound. Emerg. Dis..

