# Supplementary material for: Surveillance of Tick-Borne Pathogens (TBPs) from Wildlife in the Valle d’Aosta Region, Northwestern Italy
Source: Pathogens. 2026 Sep 21;15(9):999. doi: 10.3390/pathogens15090999 (PMC13610187; doi:10.3390/pathogens15090999)
Supplement: Supplementary file 1 [file pathogens-15-00999-s001.zip › Table S2_Supplementary Materials.pdf]

Table S2. Detection of tick-borne pathogens according to tick and host species. For each host–tick combination, the number of ticks examined (N), and the number (n) and percentage (%) of ticks positive for each pathogen with the corresponding 95% confidence interval (95% CI) are reported. Percentages were calculated using the number of ticks examined within each host–tick combination as denominator. In case of co-infections, the same tick is included in each relevant pathogen-specific column; therefore, pathogen-specific positive counts are not mutually exclusive and should not be summed.

Host	Tick species	N ticks examined	*Anaplasma* spp. n (%) [95% CI]	*B. burgdorferi* s.l. n (%) [95% CI]	*R. helvetica* n (%) [95% CI]	*R. monacensis* n (%) [95% CI]	*R. raoultii* n (%) [95% CI]	*R. slovaca* n (%) [95% CI]	*Rickettsia* spp. n (%) [95% CI]
**Roe deer**	*Ixodes ricinus*	71	2 (2.8) [0.3-9.8]	1 (1.4) [0-7.6]	8 (11.3) [5-21]	2 (2.8) [0.3-9.8]	0 (0) [0-5.1]	0 (0) [0-5.1]	1 (1.4) [0-7.6]
**Chamois**	*Ixodes ricinus*	25	2 (8) [1-26]	0 (0) [0-13.7]	0 (0) [0-13.7]	1 (4) [0.1-20.4]	0 (0) [0-13.7]	0 (0) [0-13.7]	0 (0) [0-13.7]
**Red deer**	*Ixodes ricinus*	19	1 (5.3) [0.1-26]	0 (0) [0-17.6]	1 (5.3) [0.1-26]	1 (5.3) [0.1-26]	1 (5.3) [0.1-26]	0 (0) [0-17.6]	0 (0) [0-17.6]
**Red fox**	*Ixodes ricinus*	8	0 (0) [0-36.9]	0 (0) [0-36.9]	0 (0) [0-36.9]	0 (0) [0-36.9]	0 (0) [0-36.9]	0 (0) [0-36.9]	0 (0) [0-36.9]
**European badger**	*Ixodes ricinus*	6	0 (0) [0-45.9]	0 (0) [0-45.9]	0 (0) [0-45.9]	0 (0) [0-45.9]	0 (0) [0-45.9]	0 (0) [0-45.9]	1 (16.7) [0.4-64.1]
**Red fox**	*Ixodes* spp	5	0 (0) [0-52.2]	0 (0) [0-52.2]	0 (0) [0-52.2]	0 (0) [0-52.2]	0 (0) [0-52.2]	0 (0) [0-52.2]	0 (0) [0-52.2]
**Wolf**	*Ixodes ricinus*	4	0 (0) [0-60.2]	0 (0) [0-60.2]	0 (0) [0-60.2]	0 (0) [0-60.2]	0 (0) [0-60.2]	0 (0) [0-60.2]	0 (0) [0-60.2]
**European badger**	*Ixodes* spp	3	0 (0) [0-70.8]	0 (0) [0-70.8]	0 (0) [0-70.8]	0 (0) [0-70.8]	0 (0) [0-70.8]	0 (0) [0-70.8]	0 (0) [0-70.8]
**Wild boar**	*Dermacentor marginatus*	2	0 (0) [0-84.2]	0 (0) [0-84.2]	0 (0) [0-84.2]	0 (0) [0-84.2]	0 (0) [0-84.2]	0 (0) [0-84.2]	0 (0) [0-84.2]
**Roe deer**	*Dermacentor* spp	2	0 (0) [0-84.2]	0 (0) [0-84.2]	0 (0) [0-84.2]	0 (0) [0-84.2]	0 (0) [0-84.2]	0 (0) [0-84.2]	0 (0) [0-84.2]
**Wild boar**	*Dermacentor* spp	2	0 (0) [0-84.2]	0 (0) [0-84.2]	0 (0) [0-84.2]	0 (0) [0-84.2]	0 (0) [0-84.2]	0 (0) [0-84.2]	0 (0) [0-84.2]
**Hedgehog**	*Ixodes ricinus*	2	0 (0) [0-84.2]	0 (0) [0-84.2]	0 (0) [0-84.2]	0 (0) [0-84.2]	0 (0) [0-84.2]	0 (0) [0-84.2]	0 (0) [0-84.2]
**Wild boar**	*Ixodes ricinus*	2	0 (0) [0-84.2]	0 (0) [0-84.2]	0 (0) [0-84.2]	0 (0) [0-84.2]	0 (0) [0-84.2]	0 (0) [0-84.2]	0 (0) [0-84.2]
**Roe deer**	*Ixodes* spp	2	0 (0) [0-84.2]	0 (0) [0-84.2]	0 (0) [0-84.2]	0 (0) [0-84.2]	0 (0) [0-84.2]	0 (0) [0-84.2]	0 (0) [0-84.2]
**Roe deer**	*Dermacentor marginatus*	1	0 (0) [0-97.5]	0 (0) [0-97.5]	0 (0) [0-97.5]	0 (0) [0-97.5]	0 (0) [0-97.5]	1 (100) [2.5-100]	0 (0) [0-97.5]
**Alpine ibex**	*Ixodes ricinus*	1	0 (0) [0-97.5]	0 (0) [0-97.5]	0 (0) [0-97.5]	0 (0) [0-97.5]	0 (0) [0-97.5]	0 (0) [0-97.5]	0 (0) [0-97.5]
**Beech marten**	*Ixodes ricinus*	1	0 (0) [0-97.5]	0 (0) [0-97.5]	0 (0) [0-97.5]	0 (0) [0-97.5]	0 (0) [0-97.5]	0 (0) [0-97.5]	0 (0) [0-97.5]
**Squirrel**	*Ixodes* spp	1	0 (0) [0-97.5]	0 (0) [0-97.5]	0 (0) [0-97.5]	0 (0) [0-97.5]	0 (0) [0-97.5]	0 (0) [0-97.5]	0 (0) [0-97.5]
**Beech marten**	*Rhipicephalus sanguineus*	1	0 (0) [0-97.5]	0 (0) [0-97.5]	0 (0) [0-97.5]	0 (0) [0-97.5]	0 (0) [0-97.5]	0 (0) [0-97.5]	0 (0) [0-97.5]
