# Supplementary material for: Surveillance of Tick-Borne Pathogens (TBPs) from Wildlife in the Valle d’Aosta Region, Northwestern Italy
Source: Pathogens. 2026 Sep 21;15(9):999. doi: 10.3390/pathogens15090999 (PMC13610187; doi:10.3390/pathogens15090999)
Supplement: Supplementary file 1 [file pathogens-15-00999-s001.zip › Table S1_Supplementary Materials.pdf]

Table S1. Molecular identification of Rickettsia spp. and Anaplasma spp. based on Sanger sequencing and BLAST analysis.

**Sample ID**	**Tick species**	**Host species**	**Target**	**Taxon**	**Ref. sequence accession number**	**Country of origin**	**BLAST ID%**
Rickettsia helvetica 1, I.ricinus, roe deer	*Ixodes ricinus*	Roe deer	*ompB*	*Rickettsia helvetica*	KY951985	Italy	99%
Rickettsia helvetica 2, I.ricinus, roe deer	*Ixodes ricinus*	Roe deer	*ompB*	*Rickettsia helvetica*	KY951985	Italy	100%
Rickettsia helvetica 3, I.ricinus, roe deer	*Ixodes ricinus*	Roe deer	*ompB*	*Rickettsia helvetica*	PP755204	Portugal	100%
Rickettsia helvetica 4, I.ricinus, roe deer	*Ixodes ricinus*	Roe deer	*ompB*	*Rickettsia helvetica*	KY951985	Italy	100%
Rickettsia helvetica 5, I.ricinus, roe deer	*Ixodes ricinus*	Roe deer	*ompB*	*Rickettsia helvetica*	KY951985	Italy	100%
Rickettsia helvetica 6, I.ricinus, roe deer	*Ixodes ricinus*	Roe deer	*ompB*	*Rickettsia helvetica*	KY951985	Italy	100%
Rickettsia helvetica 7, I.ricinus, roe deer	*Ixodes ricinus*	Roe deer	*ompB*	*Rickettsia helvetica*	KY951985	Italy	100%
Rickettsia helvetica 8, I.ricinus, roe deer	*Ixodes ricinus*	Roe deer	*ompB*	*Rickettsia helvetica*	KY951985	Italy	100%
Rickettsia helvetica 9, I.ricinus, red deer	*Ixodes ricinus*	Red deer	*ompB*	*Rickettsia helvetica*	KY951985	Italy	100%
Rickettsia raoultii, I.ricinus, roe deer	*Ixodes ricinus*	Roe deer	*ompB*	*Rickettsia raoultii*	PQ410726	Malaysia	99%
Rickettsia slovaca, D.marginatus, roe deer	*Dermacentor marginatus*	Roe deer	*ompB*	*Rickettsia slovaca*	AP051715	Russia	98%
Rickettsia monacensis 1, I.ricinus, roe deer	*Ixodes ricinus*	Roe deer	*ompB*	*Rickettsia monacensis*	OP729873	Spain	100%
Rickettsia monacensis 2, I.ricinus, roe deer	*Ixodes ricinus*	Roe deer	*ompB*	*Rickettsia monacensis*	OP729871	Spain	100%
Rickettsia monacensis 3, I.ricinus, roe deer	*Ixodes ricinus*	Roe deer	*ompB*	*Rickettsia monacensis*	OP729871	Spain	99%
Rickettsia monacensis 4, I.ricinus, chamois	*Ixodes ricinus*	Chamois	*ompB*	*Rickettsia monacensis*	OP729871	Spain	100%
Anaplasma phagocytophilum 1, I.ricinus, chamois	*Ixodes ricinus*	Chamois	*16S rRNA*	*Anaplasma phagocytophilum*	MZ348270	Germany	99%
Anaplasma phagocytophilum 2, I.ricinus, chamois	*Ixodes ricinus*	Chamois	*16S rRNA*	*Anaplasma phagocytophilum*	MZ348270	Germany	99%
Anaplasma phagocytophilum 3, I.ricinus, roe deer	*Ixodes ricinus*	Roe deer	*16S rRNA*	*Anaplasma phagocytophilum*	MZ348270	Germany	100%
Anaplasma phagocytophilum 4, I.ricinus, roe deer	*Ixodes ricinus*	Roe deer	*16S rRNA*	*Anaplasma phagocytophilum*	MZ348270	Germany	99%
Anaplasma phagocytophilum 5, I.ricinus, roe deer	*Ixodes ricinus*	Roe deer	*16S rRNA*	*Anaplasma phagocytophilum*	MZ348270	Germany	99%
